# Proteomic Identification of Immunodiagnostic Antigens for *Trypanosoma vivax* Infections in Cattle and Generation of a Proof-of-Concept Lateral Flow Test Diagnostic Device

**DOI:** 10.1371/journal.pntd.0004977

**Published:** 2016-09-08

**Authors:** Jennifer R. Fleming, Lalitha Sastry, Steven J. Wall, Lauren Sullivan, Michael A. J. Ferguson

**Affiliations:** 1 School of Life Sciences, University of Dundee, Dundee, United Kingdom; 2 BBI Solutions, Alchemy House, Dundee, United Kingdom; Hunter College, CUNY, UNITED STATES

## Abstract

*Trypanosoma vivax* is one of the causative agents of Animal African Trypanosomosis in cattle, which is endemic in sub-Saharan Africa and transmitted primarily by the bite of the tsetse fly vector. The parasite can also be mechanically transmitted, and this has allowed its spread to South America. Diagnostics are limited for this parasite and in farm settings diagnosis is mainly symptom-based. We set out to identify, using a proteomic approach, candidate diagnostic antigens to develop into an easy to use pen-side lateral flow test device. Two related members the invariant surface glycoprotein family, TvY486_0045500 and TvY486_0019690, were selected. Segments of these antigens, lacking N-terminal signal peptides and C-terminal transmembrane domains, were expressed in *E*. *coli*. Both were developed into ELISA tests and one of them, TvY486_0045500, was developed into a lateral flow test prototype. The tests were all evaluated blind with 113 randomised serum samples, taken from 37 calves before and after infection with *T*. *vivax* or *T*. *congolense*. The TvY486_0045500 and TvY486_0019690 ELISA tests gave identical sensitivity and specificity values for *T*. *vivax* infection of 94.5% (95% CI, 86.5% to 98.5%) and 88.0% (95% CI, 75.7% to 95.5%), respectively, and the TvY486_0045500 lateral flow test prototype a sensitivity and specificity of 92.0% (95% CI, 83.4% to 97.0%) and 89.8% (95% CI, 77.8% to 96.6%), respectively. These data suggest that recombinant TvY486_0045500 shows promise for the development of a pen-side lateral flow test for the diagnosis of *T*. *vivax* animal African trypanosomosis.

## Author Summary

African Animal Trypanosomosis presents a significant problem for agricultural development in sub-Saharan Africa and leads to large economic losses. One of the main parasites responsible is *Trypanosoma vivax*. Current diagnostic methods are either symptom-based or too costly and technologically demanding for use in endemic regions. Here, we identified *T*. *vivax* proteins selectively recognized by infected cattle sera and developed two related proteins into ELISA tests and one of these into a lateral flow test prototype. All three tests performed well when tested against randomised calf sera, suggesting good potential for the development of a pen-side *T*. *vivax* animal African trypanosomosis diagnostic device for use in endemic regions.

## Introduction

*Trypanosoma vivax* is a protozoan parasite of the genus trypanosomatidae spread primarily by biting insects. Together with *T*. *brucei* and *T*. *congolense*, it is a causative agent of Animal African Trypanosomosis (AAT) in cattle. *T*. *vivax* causes a severe version of AAT, often characterised by hemorrhagic fever as well as the more typical weight loss, fatigue and anaemia [1].

As *T*. *vivax* does not require midgut gestation within the vector it can be can be transmitted mechanically by body fluid contamination and hematophagous flies [2,3]. This has allowed the spread of the disease in South America, an area previously free from *T*. *vivax*. Over eleven million cattle are estimated to be at risk in this region [4] in addition to the 46 million cattle at risk in sub-Saharan Africa [5].

Diagnostics are limited for this parasite, relying principally on microscopy, specific antibody detection using whole parasite lysates as target antigen [6] or PCR that requires specialised equipment [7,8]. Recently, the use of heterologous soluble form variant surface glycoproteins (VSGs), such as *T*. *evansi* RoTat1.2 and *T*. *equiperdum* p64, as cross-reactive diagnostic antigens for *T*. *vivax* cattle infections has been described [9] and these may lead to new diagnostic tools. Nevertheless, at the moment, farmers mostly rely upon symptom-based diagnosis, which is complicated by the numerous other diseases with similar manifestations in the endemic regions.

With this in mind, we set out to develop a low-cost pen-side diagnostic test for *T*. *vivax* infections in cattle using lateral flow test (LFT) technology. We used the approach of identifying parasite antigens selectively recognised by cattle infection sera by proteomics, followed by recombinant protein expression in *E*. *coli* and antigen assessment by ELISA to select an antigen for LFT prototyping. This general approach has been successful for selecting diagnostic antigens for human *T*. *brucei gambiense* and cattle *T*. *congolense* infections [10–12]. One of these antigens, a recombinant invariant surface glycoprotein (rISG65-1), has been selected by the Foundation for Innovative New Diagnostics (FIND) for development of a next-generation ‘all recombinant’ LFT for human African trypanosomiasis.

Here, we report the identification, recombinant production and evaluation by ELISA of segments of two related invariant surface glycoprotein (ISG) diagnostic antigens for AAT caused by *T*. *vivax*, and the generation and evaluation of a prototype LFT with one of them.

## Materials and Methods

### Ethics statement

Rodents were used to propagate sufficient *T*. *vivax* parasites to make the detergent lysates for immunoaffinity chromatography and proteomics. The animal procedures were carried out according the United Kingdom Animals (Scientific Procedures) Act 1986 and according to specific protocols approved by The University of Dundee Ethics Committee and as defined and approved in the UK Home Office Project License PPL 60/3836 held by MAJF.

Cattle studies were approved by the ClinVet IACUC which complies with The South African National Standard: SANS 10386:2008: The care and use of animals for scientific purposes.

### Sera

All sera were provided by GALVmed. The sera used for the antigen identification by immunoprecipitation and proteomics were from four animals obtained commercially in Burkina Faso and treated prophylactically for *T*. *vivax* infections before experimental infection with *T*. *vivax*. Further sera, including 113 samples for ELISA and LFT blind testing, were obtained from Burkina Faso, Mozambique and South Africa, the latter were all from cattle raised under fly-nets. Of the 113 sera for blind testing: The Burkina Faso samples (21 sera) were from 4 calves and consisted of 4 pre-infection and 17 *T*. *vivax* post-infection sera. The Mozambique samples (20 sera) were from 2 calves and consisted of 20 *T*. *vivax* post-infection sera. The South Africa (ClinVet) samples (72 sera) were from 31 calves and consisted of 27 pre-infection and 32 *T*. *vivax* post-infection sera and 13 *T*. *congolense* post-infection sera. Strains used to infect cattle (one isolate per calf) were: In Mozambique, Y486 and IL700. In Burkina Faso, Sokoroni 18, Napie22, Komborodougou and Gondo Bengaly. At ClinVet, ILRAD560.

### IgG purification from pre- and post-infection sera

Sera were collected in Burkina Faso from four calves before and 28 days after experimental infection with *T*. *vivax*. Aliquots (250 μl) of the pre- and post-infection sera were pooled and IgG fractions were purified on protein-G Sepharose, as previously described [11, 13]. Purified IgG was coupled to CNBr-activated Sepharose 4B (GE Healthcare) at 4 mg IgG per milliliter of packed gel, according the manufacturer’s instructions.

### Preparation of *T*. *vivax* parasite lysate

Three BALB/c mice were injected with one stabilate of *T*. *vivax* ILRAD V34. After five days, infected mouse blood was harvested with citrate anticoagulant, adjusted to 5×10^4^ parasites per ml with phosphate-buffered saline (PBS) and aliquots of 0.2 ml were injected into the peritoneal cavity of 45 NMR1 mice. The mouse blood was harvested after 7 days and the parasites were purified by centrifugation, to yield a buffy coat enriched in trypanosomes, followed by DE52 ion exchange chromatography to remove white blood cells and residual erythrocytes, as described in [11, 13]. The purified trypanosomes were dissolved at 1 x 10^9^ cells.mL^-1^ in 50 mM sodium phosphate buffer, pH 7.2, 2% n-octyl-β-D-glucopyranoside (nOG) detergent containing 1X Roche protease cocktail minus EDTA as well as 1 mM phenylmethylsulfonyl fluoride (PMSF), 0.1 mM *N*-p-tosyl-L-lysine chloromethyl ketone (TLCK), 1 μg.mL^-1^ leupeptin and aprotinin. The latter protease inhibitor cocktail is efficient in protecting trypanosome proteins from proteolysis in detergent lysates for immunoprecipitation [14]. The lysate was incubated for 30 min on ice and then centrifuged at 100,000 g for 1 h at 4°C.

### Immunoprecipitation

Aliquots of *T*. *vivax* detergent lysate (10 ml) were incubated with 0.75 ml packed volume of each of the 4 mg.ml^-1^ Sepharose-IgG (infection and non-infection/control) gels, rotating for 3 h at 4°C. The gels were then packed into disposable 10 ml columns and washed with 10 ml of 10 mM Na_2_PO_4_, pH 7.2, 200 mM NaCl, 1% nOG, followed by 10 ml of 5 mM Na_2_PO_4_ pH 7.2, 1% nOG. The trypanosome proteins were eluted with 500 μl of 50 mM sodium citrate, pH 2.8, 1% nOG into tubes containing 100 μl of 1 M Tris pH 8.5 for neutralization. The eluates were further concentrated to 270 μl using a centrifugal concentrator (Millipore, 0.5 ml capacity with 10 kDa MW cut off membrane). The concentrates containing the trypanosome proteins were then transferred to low binding Eppendorf tubes and the proteins precipitated by adding 1 ml ice-cold ethanol and incubation for 24 h at −20°C.

### Proteomic protein identification

Following ethanol precipitation, the proteins eluted from the post-infection IgG and pre-infection IgG columns were dissolved in SDS sample buffer, reduced with DTT and run on a precast 4–12% Bis-Tris gradient SDS-PAGE (Invitrogen) using the MES running system. The gel was stained with colloidal Coomassie blue and equivalent regions of the infection and control lanes were cut out, reduced and alkylated with iodoacetamide and digested in-gel with trypsin. The tryptic peptides were analysed by LC-MS/MS on a Thermo Orbitrap Velos system and MaxQuant 1.4 software was used to match peptides to the predicted trypanosome protein databases [15]. Where possible, annotated gene names, or the names of homologues identified using BLASTp [16] or the protein fold/family identified with Pfam [17], were used (S1 Table). The program MaxQuant 1.4 was also used to obtain relative intensity data of the peptides recovered from the post-infection and pre-infection (control) IgG columns.

### Cloning

A DNA construct encoding residues 42–363 of TvY486_0019690 was amplified from *T*. *vivax* (strain ILRAD V34) genomic DNA using the forward and reverse primers 5’-CATATGGAGAATGAGATTGCTCGGG-3’ and 5’-GGATCCAATGCTGAGTTTGCTATTGTTAGCTGA-3’, respectively, where the underlined bases are the *Nde1* and *BamH1* cloning restriction sites. The gene TvY486__0045500, which is very similar in sequence to TvY486_0019690, could not be selectively amplified and a construct encoding residues 40–363 was instead synthesised by GenScript and optimised to avoid rare codon combinations in *E*. *coli*, unfavourable mRNA structures for protein expression and *cis* elements. The gene was obtained in a pUC vector with restriction sites (*Nde1* and *BamH1*) in place for downstream cloning. Both constructs were ligated into pCR2.1-TOPO using the TOPO TA Cloning Kit (Invitrogen) and then inserted into a pET15b-derived plasmid (Novagen) modified to include a tobacco etch virus (TEV) protease cleavage site between the N-terminal hexahistidine affinity tag and the protein sequence of interest. Recombinant gene expression of the TvY486_0045500 construct was achieved with *E*. *coli* BL21-CodonPlus (DE3) RIPL cells (Stratagene) in autoinduction medium [18] containing 50 μg mL^-1^ ampicillin and 12 μg mL^-1^ chloramphenicol. The construct for TvY486_0019690 was expressed in BL21 (DE3) Gold cells (Stratagene) in autoinduction medium containing 50 μg mL^-1^ ampicillian. Cells were cultured for 24 h at 22°C before harvesting by centrifugation (3,500 x *g*, 30 min, 4°C) the bacterial pellet was resuspended in buffer A (50 mM Tris-HCl, pH 7.5, 250 mM NaCl) containing an EDTA-free protease inhibitor cocktail (Roche).

### Protein purification of recombinant proteins

Purification was achieved using the methods described in [19]. Briefly, *E*. *coli* cells were mechanically lysed in the presence of DNAse than clarified by centrifugation (4°C, 40 min, 30,000 *g*). The proteins were captured using a 5 ml immobilised metal affinity chromatography (IMAC) (HisTrap GE Healthcare) and eluted with an imidazole gradient. Affinity tags were removed, via proteolytic cleavage (1 mg His6-TEV protease per 20 mg protein, 4 ˚C, 16 h) and the protein dialysed into buffer A (50 mM Tris-HCl, pH 7.5, 250 mM NaCl). The protease, uncleaved protein and affinity tag contaminants were removed with a further subtractive IMAC step. Final purification was achieved by size exclusion chromatography (Superdex 200 26/60) eluted with buffer A. Finally, proteins were dialysed into PBS and adjusted to at least 1 mg.ml^-1^ using 10 kDa cut-off centrifugal concentrators. All proteins were >95% pure, as judged by sodium dodecyl sulphate polyacrylamide gel electrophoresis (SDS PAGE) and Coomassie blue staining.

### Enzyme linked immunosorbent assay (ELISA)

White polystyrene Costar untreated 96 well plates were coated with 50 μl per well of target protein at a concentration of 2μg mL^-1^ in plating buffer (0.05 M NaHCO_3_, pH 9.6) then blocked with 200 μl of PBS containing 5% bovine serum albumin (BSA) and 0.1% Tween-20 overnight at 4°C. Calf sera were diluted 1:2500 in PBS containing 5% BSA, 0.1% Tween-20 and transferred in triplicate by a liquid handling device (Bio-Tek, Precision) to the ELISA plates and incubated for 1h at room temperature. After 1 h the diluted sera were aspirated and the wells were washed with PBS containing 0.1% BSA with the liquid handling device. This wash cycle was repeated 5 times. Biotinylated anti-bovine-IgG (Jackson labs) was added at dilution of 1:4000 (50 μl per well) and incubated for 1 h. Excess anti-bovine-IgG antibody was washed away (as described before) and 50 μl per well of ExtrAvidin-Horse Radish Peroxidase (HRP) at a dilution of 1:4000 was added to the plates and incubated for 1 h. The solution was aspirated and the wash steps were repeated. Finally, chemiluminescent super signal Femto substrate (Pierce) diluted 1:5 (*i*.*e*., 0.5 ml solution A, 0.5 ml solution B with 4 ml PBS) was applied to the wells at 50 μl per well and plates were read using an Envision plate reader within 5 minutes of addition of the substrate.

### Prototype lateral flow test manufacture and use

Purified recombinant TvY486_0045500 residues 40–363 (7.5 mg) were provided to BBI-Solutions, an immunoassay development and manufacturing company that has completed more than 250 lateral flow projects over the last 25 years, with manufacturing sites in Europe, USA and South Africa, and 2400 prototype LFT devices were manufactured.

The LFT is comprised of a sample pad, conjugate pad, nitrocellulose strip and top pad all attached to a backing card and housed within a plastic cassette. The sample is applied to the sample pad (followed by a chase buffer) that then flows onto the conjugate pad which contains 2 gold conjugates, a test conjugate that has p310 antigens bound to gold colloid, and a control conjugate that has an irrelevant (for the p310 detection system) antibody bound to gold colloid. The sample flows up the test strip and solubilizes the gold conjugates which then flow up the assay on to a nitrocellulose strip that has a p310 antigen line striped at the test line position. If it is a positive sample, anti-p310 antibodies within the sample will bind the p310 antigen test line and will have also bound the p310 antigen gold conjugate. This results in the p310 antigen gold conjugate being localized at the test line, due to a p310 antigen-anti p310 antibody-p310 antigen “sandwich” binding reaction. The accumulation of the gold conjugate will eventually become visible to the end user if the sample contains sufficient anti-p310 antibodies. If the sample is negative there will be no anti-p310 antibodies present and so no accumulation of gold colloid at the test line and so a test line will not appear. The sample/conjugate solution will flow past the test line and the irrelevant gold conjugate will bind at a control line and form a visible line, which is specific to the irrelevant gold conjugate, thus demonstrating successful flow of the sample and conjugates up the lateral flow assay. The sample/conjugates will then move in to the top pad of the lateral flow assay that ensures the solution flows unidirectionally along the assay.

Triplicate aliquots of 5 μl of calf sera diluted with 15 μl of PBS were added to the LFTs followed by an 80 μl of chase-buffer (PBS containing 0.05% Tween 20). Tests were discarded if upper control line was not clearly visible. After 30 min, scoring of the test bands was performed by visual comparison of freshly completed tests with a scoring card [20] (S2 Fig) and the consensus score from three devices for each serum was recorded. After reading, the nitrocellulose test strips were taken out their cases for photography.

## Results

### Selection of candidate diagnostic antigens for *T*. *vivax*

An immunoprecipitation experiment was carried out to identify candidate diagnostic antigens for *T*. *vivax*. Pooled pre-infection (day -7) and post-infection (day +28) calf sera from four animals from Burkina Faso were used to generate IgG antibody columns. Detergent lysate of bloodstream form *T*. *vivax* cells was generated from parasites recovered from mice infected with *T*. *vivax* strain ILRAD V34. Identical amounts of parasite detergent lysate were mixed with the pre-infection and post-infection Sepharose-IgG beads and proteins bound to the washed beads were eluted with low pH to break antibody-antigen interactions. The eluted samples from the pre-infection and post-infection columns were concentrated and subjected to SDS-PAGE gels for antigen separation. The pre-infection and post-infection eluates were run on separate SDS-PAGE gels to reduce potential antigen cross-contamination. The Coomassie blue stained gel lanes were cut into ten segments and each subjected to in-gel reduction and alkylation and trypsin digestion. The peptides from each gel slice were separately analysed by LC-MS/MS and the data concatenated for the pre-infection and post-infection eluate samples, respectively. These concatenated data sets were used to search the predicted protein database for *T*. *vivax* (Y486) using MaxQuant 1.4.

Many proteins (>1300) were identified in the combined data from each of the Sepharose-IgG eluates. However, the protein identification lists were sorted to select for proteins found either uniquely in the post-infection Sepharose-IgG eluate or that were >10 fold enriched in the post-infection Sepharose-IgG column eluate, as judged using the label-free quantification function in MaxQuant 1.4 [21], (S1 Table). Of the twenty-three proteins unique to the post-infection IgG eluate, all had low LC-MS/MS intensities suggesting that the immune response to these antigens, while specific, was low [10, 11]. Eleven proteins had infection: control intensity ratios >10. Two of these stood out as possible immunodiagnostic antigens, TvY486_0045500 and TvY486_0019690. These are two closely related proteins sharing 91% and 80% amino acid sequence similarity and identity, respectively. These proteins have a typical ISG domain structure, consisting of an N-terminal signal peptide, an ISG domain, a transmembrane domain and a small intracellular domain [22]. We chose to investigate these antigens because ISGs have previously proved to be good diagnostics antigens for *T*. *brucei*, *T*. *congolonse* and *T*. *evansi* [10–12].

### Expression and evaluation of recombinant *T*. *vivax* ISG antigens

Similar segments of TvY486_0045500 (amino acid residues 40–363) and TvY486_0019690 (amino acid residues 42–363), avoiding the N-terminal signal peptides and C-terminal transmembrane domains, were cloned and expressed with cleavable hexa-histidine tags in *E*. *coli*. Following nickel affinity purification and proteolytic cleavage of the hexa-histidine tags, monodisperse forms of both proteins collected from a subsequent gel-filtration purification step (and separated from aggregated material appearing at the void volume) with a final yield of 0.6 mg.L^-1^ for TvY486_0019690 and 4 mg.L^-1^ for TvY486_0045500. The predicted amino acid sequences of the two recombinant proteins are shown in (S1 Fig).

**Fig 1:**
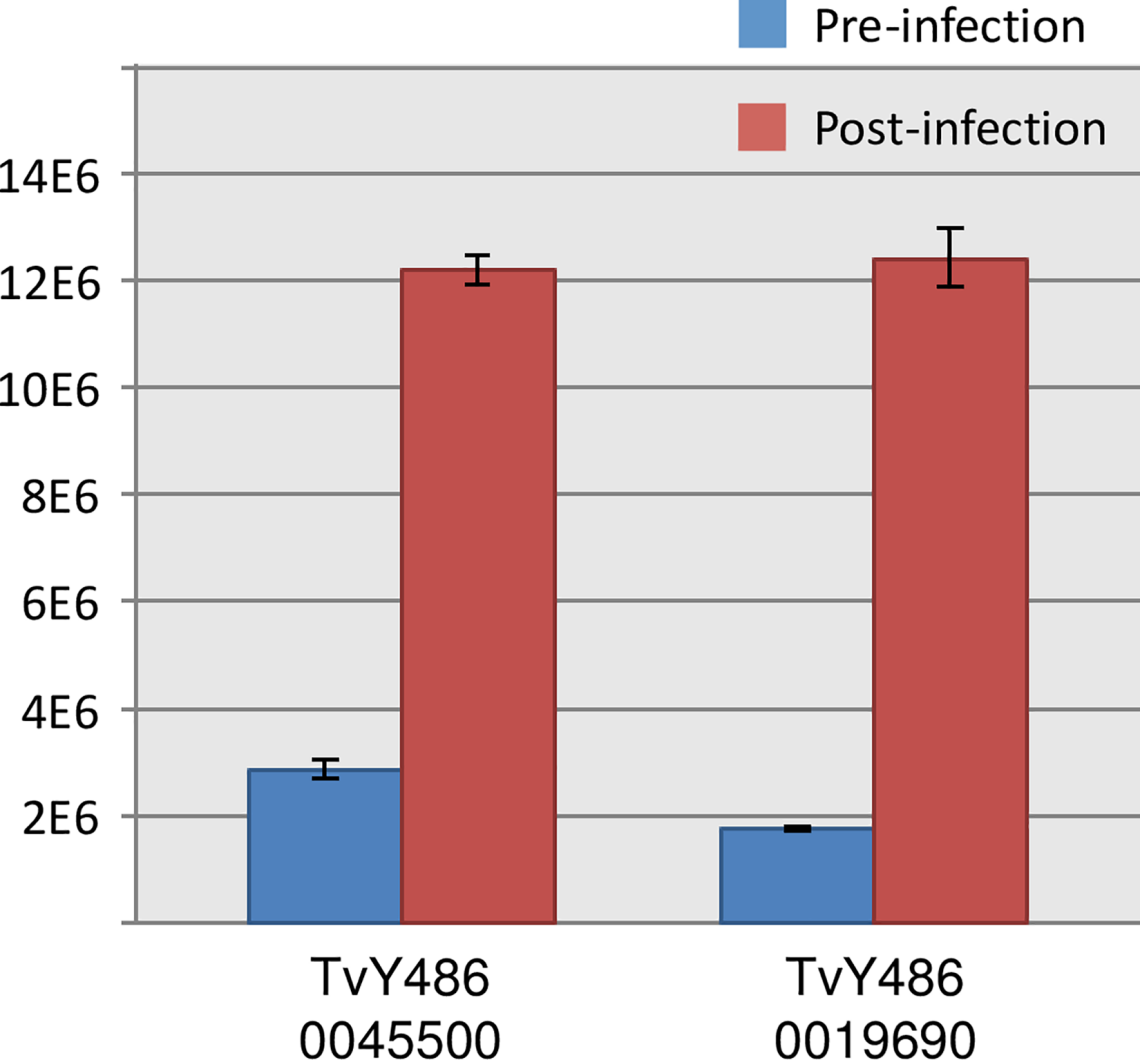
ELISA data of recombinant proteins TvY486_0045500 and TvY486_0019690 against pooled *T*. *vivax* sera from calves before (day-7) and after infection (day+28).

The two purified recombinant proteins were used to coat ELISA plates and tested with the sera of 14 calves that had been collected pre- (day -7) and post- (day +28) experimental infection with *T*. *vivax* at the ClinVet site. These data (Fig 1) indicated that both recombinant TvY486_0045500 and TvY486_0019690 coated ELISA plates could discriminate infected from uninfected sera.

TvY486_0045500 (residues 40–363) was selected for the manufacture of a prototype lateral flow device, because of its relative ease of protein expression, and was supplied to BBI Solutions (Dundee, http://www.bbisolutions.com/).

**Fig 2:**
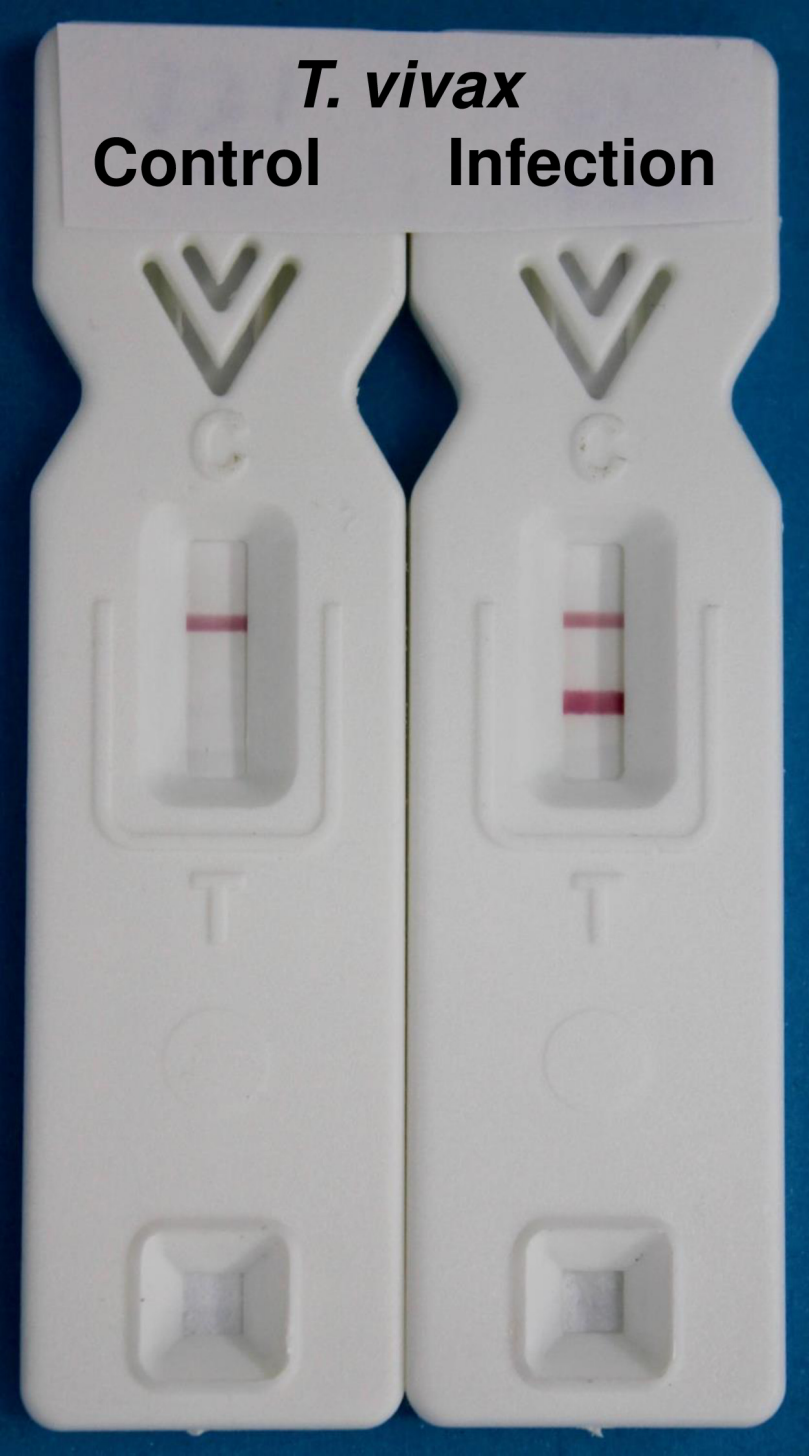
Examples of the TvY486_0045500 prototype lateral flow tests developed with control and infection sera, as indicated. Top band control line, bottom band TvY486_0045500 diagnostic line.

Both the TvY486_0045500 and TvY486_0019690 coated ELISA plates, and the TvY486_0045500 LFT prototype, were tested in triplicate with 113 randomised cattle sera provided by GALVmed. After data collection, the sera codes were broken and the data are collated in (S2 Table). Sera were classified as being either from uninfected animals or from animals that had been exposed to experimental *T*. *vivax* infection. Based on this classification, there were 69 infection (positive) and 44 control (negative) sera. Cut-off values for maximum positive/negative discrimination were determined (600,000 and 1,100,000 units for the TvY486_0045500 and TvY486_0019690 ELISA plates, respectively, and > = 2 units for the LFT) and, using these cut-offs, the sensitivity and specificity data were determined (Table 1). Representative LFT data are shown in (Fig 2) and the results of all 113 tests are shown in (S2 Fig).

**Table 1:** Sensitivity and specificity data for *T*. *vivax* ISG ELISAs and prototype LFT.

Test	Sensitivity	95% CI	Specificity	95% CI
TvY486_0045500 ELISA	94.5%	86.5% to 98.5%	88.0%	75.7% to 95.5%
TvY486_0019690 ELISA	94.5%	86.5% to 98.5%	88.0%	75.7% to 95.5%
TvY486_0045500 LFT	92.0%	83.4% to 97.0%	89.8%	77.8% to 96.6%

## Discussion

In this study, we used quantitative proteomics to identify candidate diagnostic antigens, i.e., those proteins in whole *T*. *vivax* detergent lysate that bound selectively (>10 fold more) to the immobilised IgG from calves experimentally infected for 28 days with *T*. *vivax* versus immobilised IgG from the same animals collected 7 days prior to experimental infection. In total, we found 34 candidate protein groups but rejected most of these based on the low intensities of their combined peptide ions in the LC-MS/MS analyses (we interpret low peptide intensities as an indication that only a small proportion of infection-specific IgG is directed towards their parent antigens [10,11]). The two proteins that produced the most intense peptides, and significant (around 30-fold) enrichment, were two related ISGs. We therefore focussed on these proteins and made recombinant versions in *E*. *coli* that lack the predicted cleavable N-terminal signal peptides and the predicted C-terminal transmembrane and short cytoplasmic domains. Both recombinant proteins were immunoreactive with pooled *T*. *vivax* infection sera and both antigens were subsequently tested in ELISA format against 113 randomised calf sera and found to have identical overall performance in terms of sensitivity and specificity (Table 1). Further, the Pearson coefficient between the two ELISA data sets was 0.9876, indicating that there is nothing to choose between the two antigens with respect to immunodiagnostic potential. The antigen that expressed most efficiently in *E*. *coli* (TvY486_0045500) was used to make a prototype LFT device and this was also tested blind with the same 113 randomised sera. Upon breaking the code, it became clear that we could obtain maximum discrimination between *T*. *vivax* positive and negative sera by setting the visual score cut-off at > = 2 (Fig 2) and, using these criteria, the sensitivity and specificity of the prototype LFT was similar to that of the ELISA (Table 1). These are promising performance results considering that the prototype LFT was not optimised with respect to antigen density on the test strip, antigen-gold conjugation or chase-buffer composition which, individually or collectively, should allow a reduction in background (false positive) scores of 0.5 and 1 for some sera.

Of note is that 13 of the *T*. *vivax* negative sera were from calves experimentally infected with *T*. *congolense*. None of these 13 sera gave a positive reaction with the *T*. *vivax* ISGs, either by ELISA or LFT, suggesting that the *T*. *vivax* ISGs do not routinely cross-react with *T*. *congolense* infection sera. This lack of cross-reactivity with *T*. *congolense* (and likely with other trypanosome species such as *T*. *b*. *brucei*) is expected, given that BLASTp [15] searches with the TvY486_0045500 predicted amino acid sequence does not return any significant hits against trypansomatid predicted protein databases, other than for *T*. *vivax*, [15, 22]. This is in contrast to, for example, the GM6 antigen (also recently developed into an LFT) that cross-reacts with the sera from cattle infected with multiple trypanosome species [23,24]. Both of these properties are useful, the latter with respect to making a pan-specific cattle AAT diagnostic and the former as a component of a pathogen-identifying diagnostic. It is also worth noting that BLASTp searches with the TvY486_0045500 predicted amino acid sequence does not return significant hits against any predicted protein databases, other than for *T*. *vivax*, suggesting that cross-reactivity with other non-trypanosomatid cattle pathogens is also unlikely.

## Supporting Information

S1 Fig Predicted amino acid sequences of the recombinant ISG domains used in this study. The amino acids highlighted in italics (*GHM*) come from the cleaved TEV site.
(PDF)

S2 Fig Scans of the triplicate LFT analyses of the 113 randomised sera. Randomised serum numbers (see S2 Table for details) are indicted below each group of three* LFT nitrocellulose strips that have been removed from their plastic casings. The score card used to score the intensities of the test bands is also shown. Notes: *Serum samples 29 and 108 have only duplicate LFT analyses and the samples marked ‘blank’ and ‘X’ are negative control samples developed without serum.
(PDF)

S1 Table Quantitative proteomics results. The antigens are ordered by their infection: control LC-MS/MS intensity ratios and color coded according to their absolute LC-MS/MS intensities: black bold >1000; black > 500; grey >10.
(PDF)

S2 Table Randomised serum sample numbers, sera identities and ELISA and LFT scores. (XLSX)
