# Supplementary material for: Proteomic Identification of Immunodiagnostic Antigens for Trypanosoma vivax Infections in Cattle and Generation of a Proof-of-Concept Lateral Flow Test Diagnostic Device
Source: PLoS Negl Trop Dis. 2016 Sep 8;10(9):e0004977. doi: 10.1371/journal.pntd.0004977 (PMC5015970; doi:10.1371/journal.pntd.0004977)
Supplement: S1 Fig — The amino acids highlighted in italics (GHM) come from the cleaved TEV site. (PDF) [file pntd.0004977.s001.pdf]

Figure S1. Predicted amino acid sequences of the recombinant ISG domains used in this study. The amino acids highlighted in italics (*GHM*) come from the cleaved TEV site.

>TvY486_0045500|AA_40-363
*GHM*SYENEIARALCKMGSTHRRMSMVFGVLQQRISKTDDTINGLETDLWKLKKAG
LPDEKYQEVNDKVINVTGSVSLVTNAVKVAQKKLEEFIEKVKTEHYNDHYLKLEDRK
FGESVSNCRDWATYNEETPDKLRKKLESGLKTLEAWATEESNEWEKEQKEVESDL
LSKENRNSLQYGTLHTAFKDLVKSMMVELTTVSFYMPKALEGVPGADAAVNEARK
FVVVAMANECQSVASEAAASEEKQAQCEKLNKKLQEIKEKKRQAIGGDSEGPKSS
DAKSTDATPTSSASQKVIVEEVLDSADGDELMELVQTADKPSAANNSKLS

>TvY486_0019690|AA_42-363
*GHM*ENEIARALCKMGSTHRRMSMVFGVLKQSISKTDDTINGLETDLWKLRKAGLPD
EKYQEVNDKVIDVTGSVSLVTNAVKVAQKKLEEFIKKIESGHYNDHYLQLEDRKFGE
SVSNCKNSATYNEETSEQLRKKLESGIKTLKVWAEEESKEWEKEQQDAESDLLSN
ENRNSLQYGTLHTAFKDLVNSMMVELTTVSFYMPKALEGVPGADAAVNEARKFVV
VAMANECQSVASEAAASEEKQAQCEKLNKKLQEIKEKKRQANNGDSEGPKSSDSK
SADATPTSSASQKVFVEEVLDSADSDELMELVQTADKPSSANNSKLS
