# Supplementary figures and images for: Proteomic Identification of Immunodiagnostic Antigens for Trypanosoma vivax Infections in Cattle and Generation of a Proof-of-Concept Lateral Flow Test Diagnostic Device
Source: PLoS Negl Trop Dis. 2016 Sep 8;10(9):e0004977. doi: 10.1371/journal.pntd.0004977 (PMC5015970; doi:10.1371/journal.pntd.0004977)

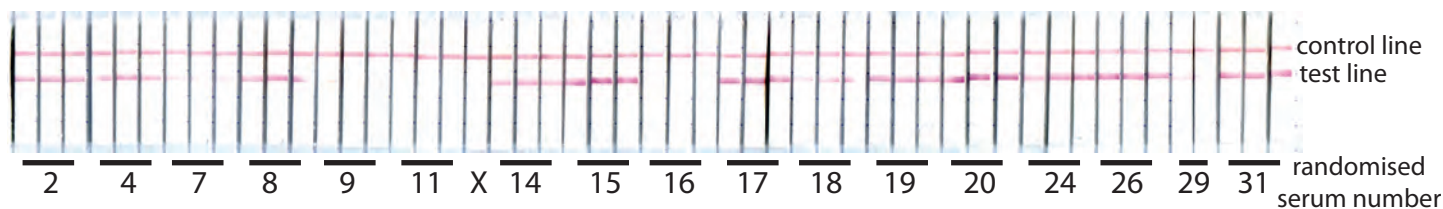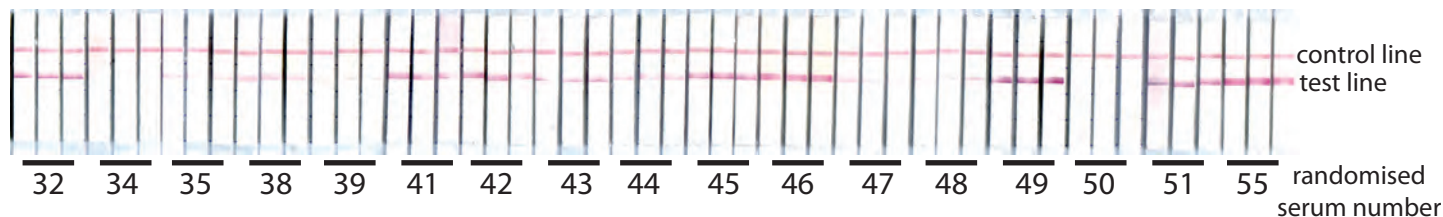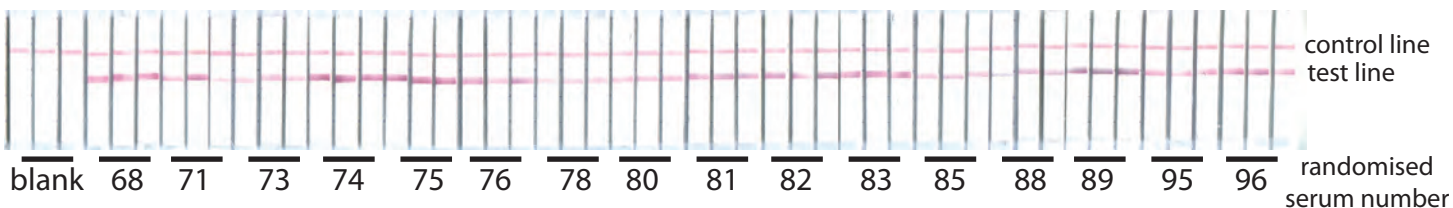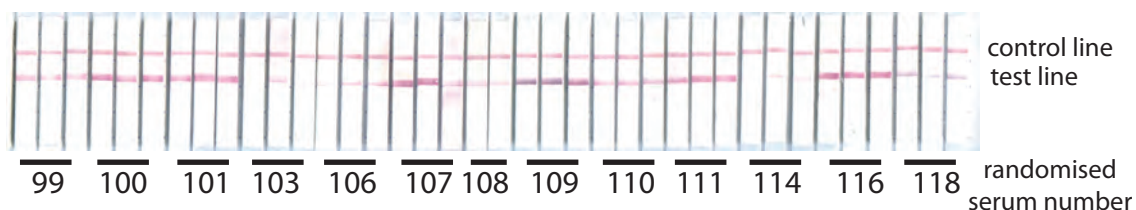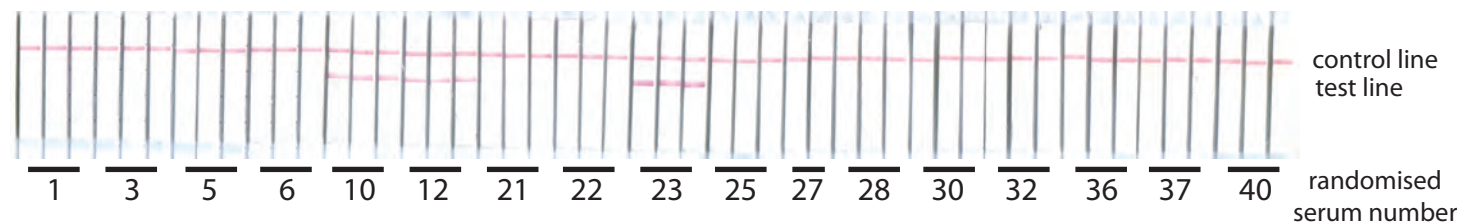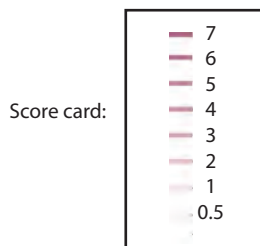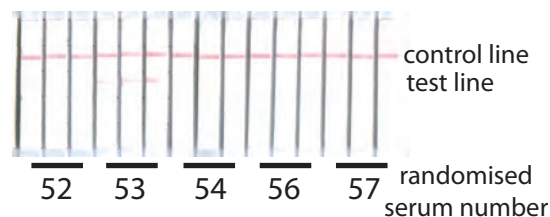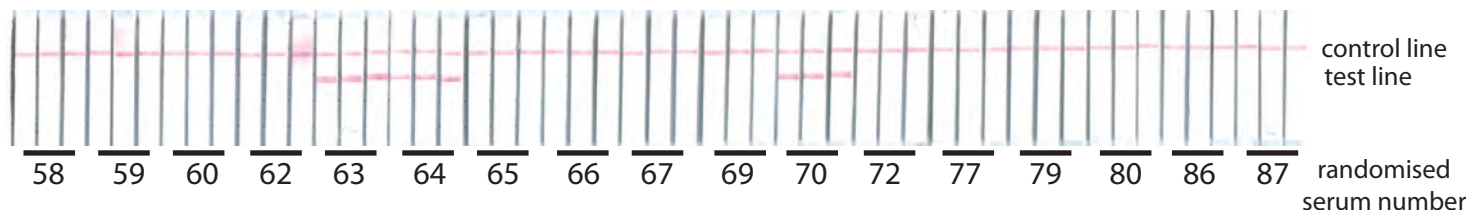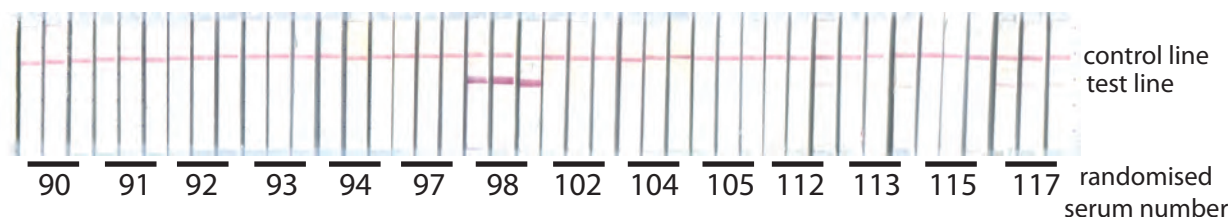

Supplement: S2 Fig — Randomised serum numbers (see S2 Table for details) are indicted below each group of three* LFT nitrocellulose strips that have been removed from their plastic casings. The score card used to score the intensities of the test bands is also shown. Notes: *Serum samples 29 and 108 have only duplicate LFT analyses and the samples marked ‘blank’ and ‘X’ are negative control samples developed without serum. (PDF) [file pntd.0004977.s002.pdf]
