# Supplementary material for: Proteomic Identification of Immunodiagnostic Antigens for Trypanosoma vivax Infections in Cattle and Generation of a Proof-of-Concept Lateral Flow Test Diagnostic Device
Source: PLoS Negl Trop Dis. 2016 Sep 8;10(9):e0004977. doi: 10.1371/journal.pntd.0004977 (PMC5015970; doi:10.1371/journal.pntd.0004977)
Supplement: S1 Table — The antigens are ordered by their infection: control LC-MS/MS intensity ratios and color coded according to their absolute LC-MS/MS intensities: black bold >1000; black > 500; grey >10. (PDF) [file pntd.0004977.s003.pdf]

Table S1: Quantitative proteomics results

The antigens are ordered by their infection : control LC-MS/MS intensity ratios and color coded according to their absolute LC-MS/MS intensities: black bold >1000; black > 500; grey >10

**Protein ID**	**Control:infected ratio**	**intensity 10^6**	**Putative name/protein family**
TvY486_0806350	74.33	37.734	
**TvY486_0045500**	**32.15**	**2875.40**	**ISG**
REV__TvY486_1013780	31.28	93.62	arm
**TvY486_0019690**	**29.46**	**1030.10**	**ISG**
TvY486_0806260	24.79	83.81	guanine deaminase
TvY486_0040570	23.77	30.10	
TvY486_0304300	16.15	74.97	5-histidyl sulfoxide synthase
TvY486_0704280;TvY486_0704300	14.02	52.13	adomet mtase, fge sulfatase
TvY486_0040800;TvY486_0013960;TvY486_0040500;TvY486_0027530;TvY486_0010260;TvY486_0027540	13.75	42.68	VSG
TvY486_0040090;TvY486_0034340;TvY486_0022920;TvY486_0034330;TvY486_0002180;TvY486_0045260;TvY486_0000*TvY486_0019690*	13.04	32.17	
TvY486_1003730	12.32	610.93	proteasome activator protein pa26
TvY486_1106220	9.71	60.63	ribosome binding
TvY486_1103390	9.55	49.96	ubiquitin-conjugating enzyme
TvY486_0906400	9.54	132.31	syntaxin
TvY486_0009580	9.29	65.29	VSG

;TvY486_0018880			
TvY486_0023330	9.25	76.89	
TvY486_0603490	8.25	150.23	kinase/hydrolase
TvY486_0007180	7.59	99.96	
TvY486_0702240	6.68	42.57	tyrosyl-tRNA synthetase,
TvY486_0041380 ;TvY486_0038160	6.56	12.50	
TvY486_0040150	6.47	16.71	
TvY486_1106950	6.46	852.61	M17 aminopeptidase
TvY486_0010*TvY486 _0019690*	6.32	9.46	
TvY486_1109080 ;TvY486_1109070	5.14	410.26	ribonuclease II-like protein
